# Developmental interneuron subtype deficits after targeted loss of *Arx*

**DOI:** 10.1186/s12868-016-0265-8

**Published:** 2016-06-10

**Authors:** Eric D. Marsh, MacLean Pancoast Nasrallah, Caroline Walsh, Kaitlin A. Murray, C. Nicole Sunnen, Almedia McCoy, Jeffrey A. Golden

**Affiliations:** Department of Pediatrics, Children’s Hospital of Philadelphia, Philadelphia, PA USA; Department of Pathology, Children’s Hospital of Philadelphia, Philadelphia, PA USA; Division of Child Neurology, Children’s Hospital of Philadelphia, Room 502E, Abramson Research Building, 3615 Civic Center Boulevard, Philadelphia, PA 19014 USA; Department of Neurology, Perelman School of Medicine at the University of Pennsylvania, Philadelphia, PA USA; Department of Pathology, Perelman School of Medicine at the University of Pennsylvania, Philadelphia, PA USA; Department of Pathology, Brigham and Women’s Hospital, Harvard Medical School, 75 Francis St., Boston, MA 02115 USA

**Keywords:** Interneuron, Development, Migration

## Abstract

**Background:**

Aristaless-related homeobox (*ARX*) is a paired-like homeodomain transcription factor that functions primarily as a transcriptional repressor and has been implicated in neocortical interneuron specification and migration. Given the role interneurons appear to play in numerous human conditions including those associated with *ARX* mutations, it is essential to understand the consequences of mutations in this gene on neocortical interneurons. Previous studies have examined the effect of germline loss of *Arx,* or targeted mutations in *Arx*, on interneuron development. We now present the effect of conditional loss of Arx on interneuron development.

**Results:**

To further elucidate the role of *Arx* in forebrain development we performed a series of anatomical and developmental studies to determine the effect of conditional loss of *Arx* specifically from developing interneurons in the neocortex and hippocampus. Analysis and cell counts were performed from mouse brains using immunohistochemical and in situ hybridization assays at 4 times points across development. Our data indicate that early in development, instead of a loss of ventral precursors, there is a shift of these precursors to more ventral locations, a deficit that persists in the adult nervous system. The result of this developmental shift is a reduced number of interneurons (all subtypes) at early postnatal and later time periods. In addition, we find that X inactivation is stochastic, and occurs at the level of the neural progenitors.

**Conclusion:**

These data provide further support that the role of *Arx* in interneuron development is to direct appropriate migration of ventral neuronal precursors into the dorsal cortex and that the loss of *Arx* results in a failure of interneurons to reach the cortex and thus a deficiency in interneurons.

## Background

Aristaless-related homeobox (ARX) is a paired-like homeodomain transcription factor that functions primarily as a transcriptional repressor and has been hypothesized to be important for interneuron specification and migration [1–6]. Patients harboring a mutation in *ARX* present with a spectrum of neurological disorders ranging in severity from lissencephaly with ambiguous genitalia (XLAG syndrome) to those with isolated intellectual disabilities (ID) [7–9]. All patients with *ARX* mutations have some degree of intellectual disability, and almost all develop epilepsy during early childhood. A relatively consistent genotype-phenotype correlation exists with *ARX* mutations: protein truncation or homeodomain loss of function results in both ID and epilepsy with severe structural defects, while point mutations outside the homeodomain or expansions in one of the 4 alanine tracts leads to ID and epilepsy phenotypes alone [5, 7]. Mice with mutations in *Arx*, such as an alanine tract expansion or specific point mutations, reproducibly model many of these phenotypes including a striking developmental epilepsy [10, 11]. Additional studies suggest the epilepsy phenotype is largely or entirely the result of a cortical interneuron defect [2, 12].

This “interneuronopathy hypothesis” proposes that a developmental deficiency of inhibitory cortical interneurons caused by defects in proliferation, specification, differentiation and/or migration, results in epilepsy [13, 14]. Our previous findings are consistent with this hypothesis; the epilepsy phenotype was observed in mice in which *Arx* was conditionally abrogated from interneurons (the *Arx*^−*/Y*^*; Dlx5/6*^*cre*-*IRES*-*GFP(CIG)*^ cross) but not when *Arx* is selectively excised from dorsal progenitors of projection neurons (*Arx*^−*/Y*^*; Emx*^*CRE*^ cross) [2, 12]. These mouse models supports the interneuronopathy hypothesis, however none of our previous studies investigated the full alteration in interneurons in the *Arx* mice.

Although previous studies (including from our labs) on both *Arx*^−*/y*^ and *Arx*^−*/Y*^*; Dlx5/6*^*(CIG)*^ mice have a cortical interneuron deficit, many interneurons still persist in the neocortex [2, 8, 11]. Our previous studies have evaluated adult cortical and hippocampal interneurons, but did not completely characterize the various interneuron subtypes and how interneurons change through development [2]. Hence, identifying the subpopulations of interneurons that persist and how they function remains an important question that may help in understanding not only the pathobiology underlying *ARX* mutations, but also potentially other forms of developmental epilepsies with intellectual disabilities.

Interneurons can be subdivided by morphology, neurochemistry and electrophysiologic properties [15–17]. While a number of transcription factors have been identified as critical to establishing the diversity of interneuron subtypes, the specific role of these transcription factors in delineating interneuron subpopulations is incompletely understood. The *Dlx* transcription factors, expressed early in the interneuron lineage, appear to be essential in controlling interneuron fate [18, 19]. Multiple other transcription factors (for example *Nkx2.1*, *Lhx6*, and *Sox6*) have also been identified as necessary for interneuron subtype determination, refinement of the lateral, medial, and caudal ganglionic eminence populations, and migration to the olfactory bulb, striatum, and cortex (For review see [15, 20, 21]). Though it is clear that loss of *Arx* alters interneuron development, and that *Arx* function is primarily, though not exclusively, downstream of the *Dlx* transcription factors, the ultimate role of *Arx* in this process is incompletely understood [22].

To further define the interneuron populations impacted by loss of *Arx* we analyzed interneuron subtypes through development in mice conditionally mutant for *Arx* in interneurons. Our data, which expands our previous studies [2], indicate that early in development, instead of a loss of ventral precursors, there is a shift of these precursors to more ventral locations, a deficit that persists in the adult nervous system. The result of this developmental shift is a reduced number of all subtypes of interneurons at early postnatal and later time periods. These data suggest that ARX is important for the dorsally directed migration of most interneurons into the cortex but that ARX appears to have a limited role in interneuron specification.

## Methods

### Generation/care of mice

All experiments were approved by the Children’s Hospital of Philadelphia Animal Care and Use Committee. The animals were kept in standard mouse cages, on a 12-h light/dark cycle, and allowed free access to food and water. The care and husbandry of the mouse colony followed previous descriptions [1, 2]. For this study double or single floxed females (*Arx*^*fl/fl*^ and *Arx*^*fl/*+^) were crossed to *Dlx5/6*^*CIG(cre*-*IRES*-*GFP*)^ males [23]. In addition, the *Dlx5/6*^*CIG*^ males were mated with tdTomato reporter mice (*tdTomato*^*fl*-*stp/fl*-*stp*^) (from Jackson Labs- Ai9(RCL-tdT)- stock number 007909) to generate *Dlx*^*CIG*^*;tdTomato*^+*/*−^ males. These males were crossed to *Arx*^*fl/fl*^ females to generate animals that had tdTomato expression in *Dlx5/6* progenitors either with or without *Arx*. *Arx*^*fl/*+^ or ^*Y*^, *Dlx5/6*^*CIG*^, and the *CD1* and *C57/Bl6* background genotypes were all considered controls.

### Gross anatomy and histology

P14 and adult Animals were deeply anesthetized and then transcardially perfused with PBS followed by 4 % paraformaldehyde. The brains were harvested, weighed, further fixed in 4 % paraformaldehyde overnight at 4 °C, and then transferred to PBS. Each whole brain was carefully viewed to characterize gross morphologic differences. Four tissue blocks were prepared: (1) posterior boundary of the olfactory bulbs to 2 mm posterior on the frontal cortex, (2) frontal cortex to 2 mm anterior to the optic chiasm, (3) 2 mm anterior to 1 mm posterior to the chiasm, and (4) 1 mm posterior to the chiasm to the occipital cortex. The four tissue blocks were embedded in paraffin and sections cut at 4 µm for histology and immunohistochemical studies. One slide per region was stained with hematoxylin and eosin.

### Immunohistochemistry

Immunohistochemistry was performed using a standard protocol as previously performed in our lab with antibodies against interneuron markers (Calb-calbindin, Calr-calretinin, Parv—parvalbumin, NPY—Neuropeptide Y, and SST—somatostatin), GFP (anti-GFP) and Arx (see Table 1 for manufacture information and staining parameters) [2, 3, 12]. Antigen retrieval was performed by microwaving (2 min on high, 3 min at medium power) the slides in citric acid buffer. Secondary antibodies are presented in Table 1.

**Table 1:** Primary and secondary antibodies

Primary antibody	Species	Company	Dilution	
Calretnin	Rabbit	Swant	1:100	
Arx	Rabbit	Gift from Kunio Kitamura	1:1000	
Calbindin	Rabbit	Swant	1:500	
SST (somatostatin)	Rabbit	ABCAM (Epitomics)	1:50	
Nkx2.1				
GFP	Rabbit	Cell Signaling	1:800	
NPY	Rabbit	Millipore	1:500	
Parvalbumin	Mouse	Millipore	1:100	

Secondary antibody	AB	Species	Company	Dilution
Biotin	Anti rabbit	Donkey	Jackson Immuno	1:500
Biotin (H+L) & (Fab’)	Anti goat	Donkey	Jackson Immuno	1:500
Biotin	Anti mouse	Goat	Jackson Immuno	1:500
FITC	Anti rabbit	Goat	Jackson Immuno	1:50
TRITC	Anti rabbit	Donkey	Jackson Immuno	1:50

### In situ hybridization

e14 and >P30 mice were processed as described above and in situ hybridization performed as previously described [12]. Briefly, all slides were baked at 65 °C, post-fixed in 4 % PFA, washed in PBS with 0.1 % Tween-20 (PBT) and acetylated for 10 min in 0.1 M TEA (1.86 % triethanolamine, 0.4 % 10 N NaOH, 0.5 % acetic anhydride, and water). The glutamate decarboxylase 67 (GAD67) probe (courtesy of Dr N. Tillakaratne; Erlander et al. [24]) was added at 1:1000 in hybridization solution and incubated overnight at 65 °C. Slides were then washed and incubated in MABT pH 7.5 (100 mM maleic acid, 150 mM NaCl, 0.1 % Tween-20, and water), blocking agent, and 20 % goat serum for 1 h at room temperature. Next, the slides were incubated overnight at 4 °C in MABT with 2 % blocking reagent and 5 % goat serum and anti-DIG antibody 1:2500 (Roche, 11093274910). The reacted slides were then washed and incubated with 1 mL of BM Purple (Roche, 11442074001) in the dark, overnight at room temperature. Lastly, the slides were washed with NTM and PBS, fixed in PFA, dehydrated to 100 % ethanol, stepped to Xylene and mounted with Permount (SP15-500, Fisher Scientific).

### Imaging and cell counting

The tissue was visualized using a Leica DMR microscope (Leica Microsystems, Bannockburn, IL) equipped with epifluorescence and light microscopy. The images were acquired with an Orca digital camera (Hamamatsu, Hamamatsu City, Japan) and processed in Image Pro^®^ software for cell counting. Sections from the most anterior block (in M1 and lateral neocortex), sections from the optic chiasm block (S1 and lateral neocortex), and sections through the hippocampus (in sagittal section) were chosen for cell counting. Two 10× magnification images were taken and stitched together to produce a continuous image from the ventricular to pial surface. Fully labeled cells were measured and numbered from a reference point on the ventricular surface in the middle of the acquired image. This method insures accurate measures of the distance from the ventricular surface (layer and location information) as well as numbers of cells (total quantitative information). All counting was performed blind with respect to the genotype by two individuals. Counts from sections were averaged for each animal and then compared between genotypes.

### Statistical analysis

All statistics were performed in Matlab using the statistics toolbox. A first test of differences between groups was performed using an N-way ANOVA. Each genotype was considered as an independent variable. For all ANOVA calculations, the main effect had 4 degrees of freedom based on 5 genotypes: (1) wild type (*Bl6/C57*), (2) *Arx*^*fl*/+^ or ^*y*^ (3) *Dlx5/6*^*CIG*^ (4) *Arx*^−*/*+^;*Dlx5/6*^*CIG*^ (female Hets); and (5) *Arx*^−*/y*^;*Dlx5/6*^*CIG*^ (ARX-/Y). The level of significance was set at α = 0.01. As there was no difference between the control genotypes in the cell count analysis (data not shown), the N-way ANOVA was repeated with the *Arx*^*fl*/+^ or ^*y*^, *Dlx5/6*^*CIG*^ and wildtypes pooled as the control group and compared to the *Arx*^−*/*+^; *Dlx5/6*^*CIG*^ (female Hets); and *Arx*^−*/y*^; *Dlx5/6*^*CIG*^ (ARX-/Y males). After calculation of the ANOVA, post hoc analysis of sub group differences was performed in Matlab using the ‘anovarep’ function that performs a Holm-Sidak test for multiple comparisons to determine if subgroups are different (http://www.mathworks.com/matlabcentral/fileexchange/18746). In addition, the combined control group was independently compared to the Arx^−*/*+^; *Dlx5/6*^*CIG*^ (female heterozygous mice) in the adult animals with Student t-tests.

For the embryonic time points, due to the many densely packed cells, which posed significant difficulties counting individual cells, we used a ranking method to quantify the differences between the genotypes. For this method, we printed out and presented the photo-micrographs of each of the regions of interest (ROI) to 6 individuals who were blinded to the genotype. Raters were asked to rank the photo-micrographs in order of number of labeled cells in the region of interest with the image showing the greatest number of labeled cells ranked 1, and the image showing the fewest labeled cells ranked last. The rankings were averaged across all individuals. Then the mutant and wild type group ROI averages were compared using a three way ANOVA (see above for details).

For quantification of embryos and pups born at different ages, we performed a Chi-square test with the expected ratio being normal Mendelian ratios. This was compared to the actual ratios of fetuses harvested or pups born and survived to P14.

## Results

**Fig. 1:**
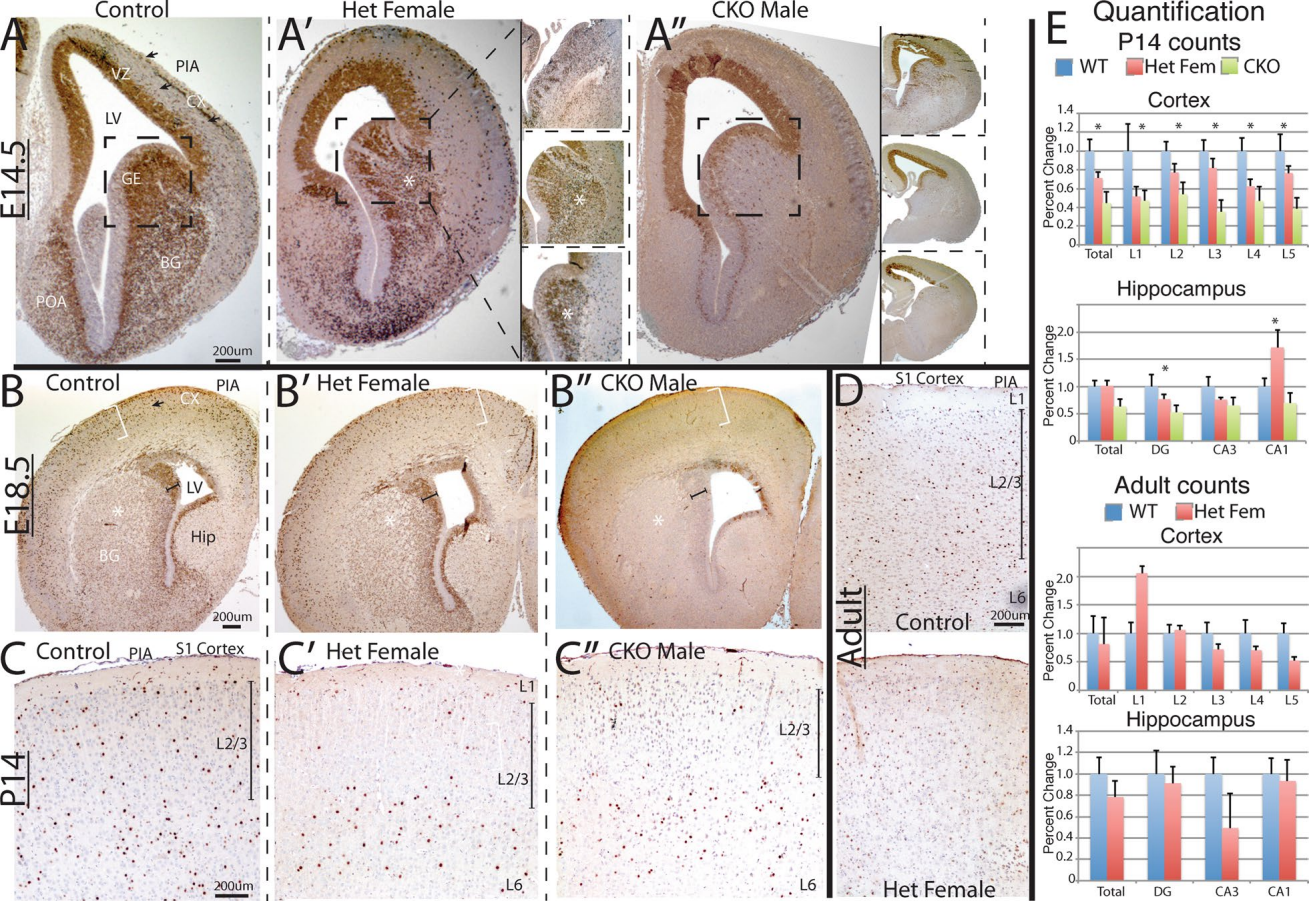
Arx expression across the ages in control and *Arx* ^−*/x*^ and *Arx* ^−*/y*^ *;Dlx5/6* ^*CIG*^ mice. **A**–**A**″ e14.5 embryos in coronal plane. **A** control, **A**′ female het, **A**″ male CKO. Immunohistochemistry for Arx reveals near complete loss of Arx in the ventral pallium, in both VZ, SVZ and developing basal ganglia (**A**″), but persistence in the cortical VZ. There is near complete loss of Arx positive cells in the developing cortex as well in the male CKO (**A**″). Even with loss of *Arx*, the anatomy of the ventral pallium is unchanged (*boxed areas* in **A**″). **A**′ *insets* (from area around *asterisk* in main image) reveals a spectrum of X inactivation patterns and variability of expression of *Arx* in 4 representative examples of heterozygous females at e14.5. Only the GE is highlighted in *each inset*. Stripes of clonal expansion of both Arx absence and presence are observed emanating from the VZ. **A**″ *insets*—four representative examples demonstrating the variability of loss of Arx in CKO males; from moderate loss (*top inset*) to complete loss (*bottom inset*). *Arrows* in **A** highlight migrating interneurons. **B**–**B**″ Representative e18.5 coronal images. **B** Control, **B**′ female het, **B**″ male CKO. At 18.5 there are many fewer cell in the *Arx* ^−*/y*^ *;Dlx5/6* ^*CIG*^ than in the wildtype, including the dorsal cortex (*white brackets*), ventral BG (*asterisk*) and VZ of GE (*capped black line*). There are an intermediate number of cells in both locations in the female heterozygous animals (**B**′). Again note anatomy of developing BG is unchanged in all three genotypes. **C**–**C**″ Representative P14 coronal images. **C** Control, **C**′ female het, **C**″ male CKO. Arx staining illustrates that the persistent Arx positive cells in the surviving *Arx* ^−*/y*^ *;Dlx5/6* ^*CIG*^ mice (**C**″). In this animal the cells were mostly in the lower layers but there was variability in the 4 mutants so this was not statistically significant. A similar pattern was also found in many of the heterozygous females (**C**′) whereas the wildtype brains have cells dispersed throughout the cortex (**C**). **D** Representative adult coronal images. *Upper panel* control, *lower panel* female het. No significant differences are noted. **E** Quantification of Arx cell numbers in cortex and hippocampus at P14 and in adult animals across all layers and hippocampal regions. *Upper panels* are counts from P14 animals. Total is the sum of the counts of all layers. L1–L5 are five equally spaced regions that correspond roughly to cortical layers (see “Methods”). *Asterisk* signifies statistical differences. *Lower panels* are counts from adult animals. *LV* lateral ventricle, *SVZ* subventricular zone, *VZ* ventricular zone, *BG* basal ganglia, *GE* ganglionic eminence, *Cx* developing cortex. *Hip* hippocampus. *L1* cortical layer 1, *L2/3* cortical layers 2 and 3, *L6* cortical layer 6

Previous studies querying *Arx* function have shown an important role in interneuron development and suggested a role in both migration and cell differentiation [8, 22, 25, 26]. These studies employed a complete knockout of *Arx* to assess the ventral forebrain role of *Arx*, possibly complicating the analysis as dorsal progenitor function is also altered. Hence, to analyze the cell autonomous role of *Arx* in interneuron development we generated *Arx* conditional mutant mice with near complete loss of *Arx* from the ventral forebrain at e14.5 (embryonic day 14.5) in *Arx*^−*/Y*^; *Dlx5/6*^*CIG*^ males (Fig. 1A–A″, insets a″). In females there were radially oriented streams of Arx within and emerging from the GE ventricular zone, which varied dramatically between embryos (Fig. 1A′, insets). This apparent loss of some *Arx* cells dissipated when the *Arx* cells migrated into the subventricular and mantle zones of the GE and mixed with the Arx positive cells (Fig. 1A′, asterisk, insets). Loss of Arx was more variable in males than females (Fig. 1A′, A″, insets). The striated expression in *Arx*^−*/*+^; *Dlx5/6*^*CIG*^ female animals (Fig. 1A′, boxed areas) occurred on some level in all females and appears to be clonal proliferations of *Arx* positive or negative cells that migrate radially away from the VZ of the GE. The striation occurs in the VZ of the GE suggesting this is the site of random X-inactivation in the ventral forebrain.

**Fig. 2:**
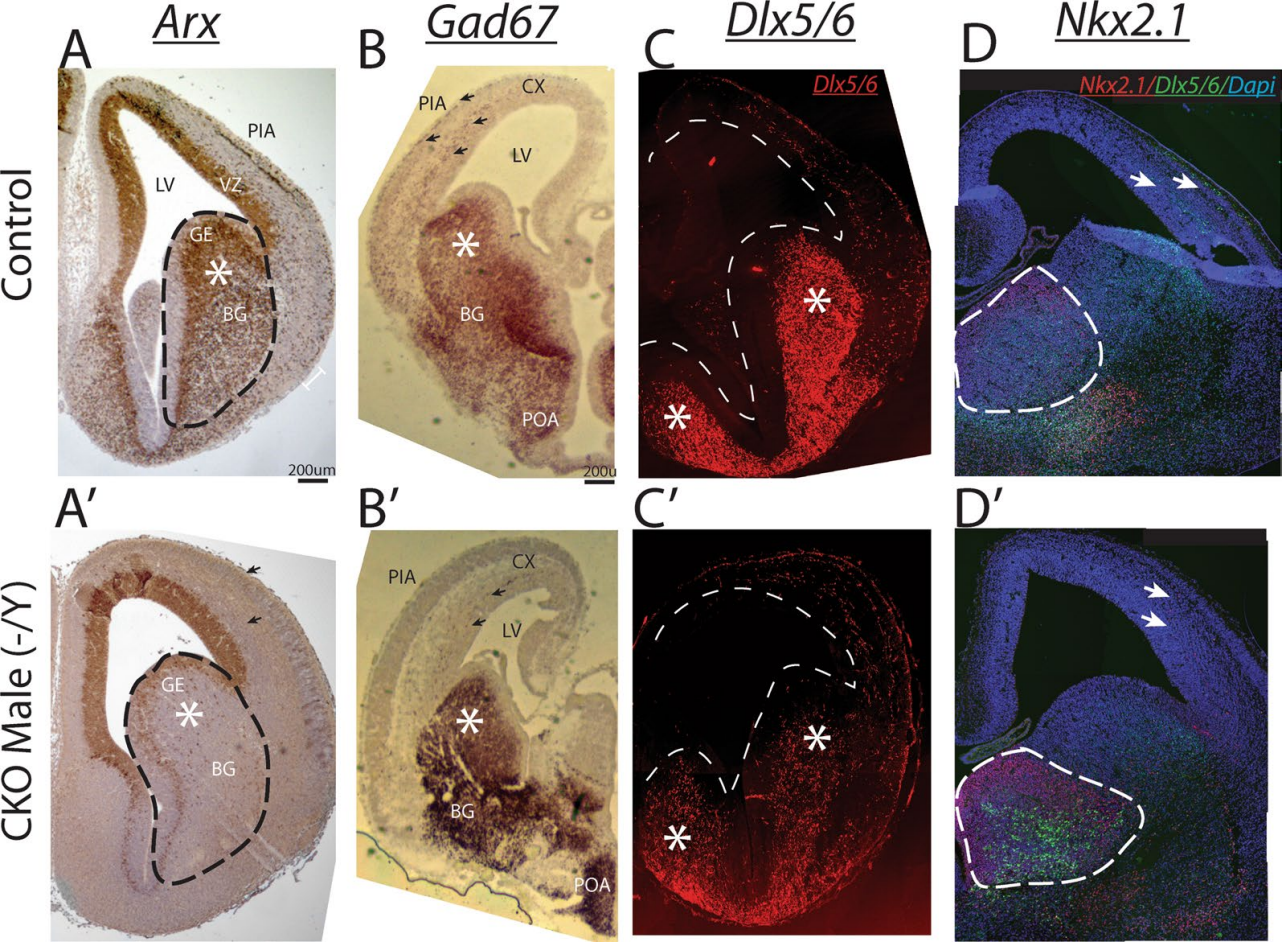
Interneuron migration is altered, but progenitor domains are not at e14.5. For all *panels*—*upper row* is control sections, *lower row* is male CKO. **A** Re-presenting A from Fig. 1 for reference and highlighting anatomy of ventral foerbrain. **B** In situ hybridization for *Gad67* demonstrates normal appearance of cells in the ventral pallium (*asterisk*) and substantial decrease in migrating cells (*arrows*) in the cortex with remaining cells migrating in the SVZ (*arrows*). **C**–**C**′ Further confirmation of preservation of ventral interneuron progenitor pool with *Dlx5/6*—tdTomato fluorescence being unchanged between wildtype and mutant embryos. Anatomy of section is highlighted by *dashed white outline.* **D**–**D**′ Co-labelling of *Dlx5/6*-GFP in *green*, Nxk2.1 in *red* and nuclei in *blue* (DAPI labeling) shows normal *Nxk2.1* expression and normal *Dlx5/6* expression, again with reduction of neurons migrating to cortex. Again note with DAPI labeling that anatomy of ventral forebrain is unchanged in the male CKO. *LV* lateral ventricle, *SVZ* subventricular zone, *VZ* ventricular zone, *BG* basal ganglia, *GE* ganglionic eminence, *Cx* developing cortex

While Arx was clearly lost from the GE precursors, the regional anatomy of the GE at e14.5 was unchanged, suggesting that there was no loss of cells, but only a loss of *Arx* (for example compare ventral forebrain in Fig. 1A, or Fig. 2). To confirm this observation we performed in situ hybridization for *Gad67* on wildtype and mutant embryos. We observed that the overall number and location of the Gad67 positive cells was unchanged (Fig. 2B, B′). To further verify that *Dlx* progenitor cells are not lost with abrogation of *Arx* in the Arx^−/y^ males, we fate mapped Dlx5/6 positive cells by crossing *Dlx5/6*^*CIG*^ mice to tdTomato reporter mice and then crossed the F1 generation to *Arx*^*fl/fl*^ animals. At e14.5, there was no change in the ventral expression of fate mapped *Dlx5/6* progenitors (Fig. 2C, C′). Finally, a secondary check on the regional expression pattern was performed by determining if expression of *Nxk2.1* was altered in the *Arx*^−*/y*^ males by performing immunohistochemistry on the e14.5 sections from the *Arx*^*fl/lf*^ to *Dlx5/6*^*CIG*^ cross. As expected with no change in Gad67 expression, there was no change in the expression of the *Nkx2.1* as well. Staining for the intrinsic GFP in these animals also showed no change in *Dlx5/6* expression, confirming the tdTomato data (Fig. 2C, C′). These data support the previous work of Collombat and colleagues which suggests *Arx* is not important for interneuron progenitor maintenance or fate specification as all cells remain and differentiate into Gad positive neurons [8, 26].

Prior reports by Kitamura and Collombat had demonstrated that the interneurons that did migrate to the cortex were found primarily in the SVZ and not in the IZ and MZ [8, 25]. In these studies, *Arx* was also removed from the dorsal VZ raising the possibility that interneuron development was indirectly influenced by the dorsal loss of *Arx*. Our data support and expand these previous reports; we find that in *Arx*^−*/*y^; *Dlx5/6*^*CIG*^ mice, very few cells migrate out of the GE and then primarily into the SVZ migratory stream of the dorsal cortex leading to very few cells present in the developing cortex (Figs. 1A, 2). The migrating cells appear to enter the cortex appropriately as we found no statistically significant shift to lower layers at P14 in surviving *Arx*^−*/Y*^; *Dlx5/6*^*CIG*^ mice (Fig. 1E, top graph). The ultimate fates of these cells, while lower in number also appeared to be unchanged as we saw a normal ratio of interneuron subtypes when comparing the counts of cells in different sections (e.g. Calb WT-38.2 % of *Arx* positive cells present vs 24.8 in *Arx*^−*/y*^; Calr WT-4.3 % vs. *Arx*^−*/y*^-1.8 %; Npy WT-22.8 vs 19.9 %).

Similar to our previous findings, we observed a decreased number of *Arx*^−*/y*^; *Dlx5/6*^*CIG*^ progeny including numerous perinatal and juvenile deaths [1, 2]. To quantify the prenatal and postnatal loss of mutants, we genotyped 12 litters at e14.5. Genotyping performed at e14.5 gave Mendelian ratios of offspring (27:24:49 %, n = 12 litters, for animals of genotypes *Arx*^−*/Y*^*; Dlx5/6*^*CIG*^:*Arx*^−*/X*^*; Dlx5/6*^*CIG*^:*Arx*^*Fl/X*^ or *Arx*^*Fl/Y*^). The number of *Arx*^−*/Y*^; *Dlx5/6*^*CIG*^ animals that were present at e18.5 was similar to e14.5. However, when litters were allowed to proceed to birth and genotyping was performed postnatally, we saw a reduction in survival at P12-14 (only 6 *Arx*^−*/Y*^*; Dlx5/6*^*CIG*^ males out of 15 litters) and at the time of weaning; only three *Arx*^−*/Y*^*; Dlx5/6*^*CIG*^ mice remained in 15 litters (0.02 % of offspring, p < 0.001 Chi-squared test). While the definitive cause of these postnatal deaths remains uncertain, we believe neurological dysfunction is likely given both the inability to smell/feed and seizures [2, 12]. We do know that the perinatal death is not from the pancreatic defect as loss of *Arx* in the pancreas during development does not result in perinatal death [28] as previously postulated [29].

To further determine the role of *Arx* in later interneuron development and interneuron subtype specification, we traced the surviving Arx positive cells in males and heterozygous females, throughout development. Staining for Arx in cortical and hippocampal sections from *Arx*^−*/Y*^, *Arx*^−*/X*^, and wildtype littermates demonstrated that a number of Arx positive cells remained after birth (Fig. 1C, D). At e18.5, P14, and in adult mice there was significant variability in the number of Arx positive cells similar to e14.5 mice. Arx expression at e18.5 was similar to that seen in e14.5 *Arx*^−*/Y*^ animals, with both demonstrating almost complete loss of *Arx* in the SVZ and developing basal ganglia and rare scattered cells in the cortex (Fig. 1A″–B″). e18.5 *Arx*^+*/*−^ brains also showed considerable variability, with some brains having normal Arx expression in the cortex and remnants of SVZ in the GE and some with a clear reduction (Fig. 1B″ for example of almost complete loss of Arx). At P14, in the few surviving *Arx*^−*/Y*^ animals, there was a clear reduction in the total number of Arx positive neurons, more prominent in upper cortical layers with some preservation in deeper layers (Fig. 1C″, e for graph of counts; p < 0.004 for whole cortex). While a statistical difference was found in all layers, the lower layers did not have greater differences then the upper layers, though in some animals there appeared to be more cells in the lower layers (Fig. 1C′, C″). The P14 *Arx*^+*/*−^ mice showed moderate variability with regard to cortical interneuron numbers. Some animals had a normal number and distribution of interneurons while others showed a reduction in the number of interneurons (Fig. 1C″, E). Hippocampal images were also significantly different, although less dramatic then in the cortex of *Arx*^−*/Y*^ mice at P14 (Fig. 1E, second graph, p = 0.037, images not shown).

We interpreted the two adult *Arx*^−*/y*^ animals where Arx labeling appeared normal (data not shown) to be either incomplete excision of Arx or mosaicism, with loss of Arx in the tail but preservation in the brain. Because of this possible mosaicism and few surviving animals, we did not count the adult *Arx*^−*/y*^ male animals. In adult heterozygous females, there again was variability with some animals having normal expression and others reduced (Fig. 1D, E with the averages being similar; p = 0.12) although differences were noted in the hippocampus (p = 0.013, Fig. 1E).

**Fig. 3:**
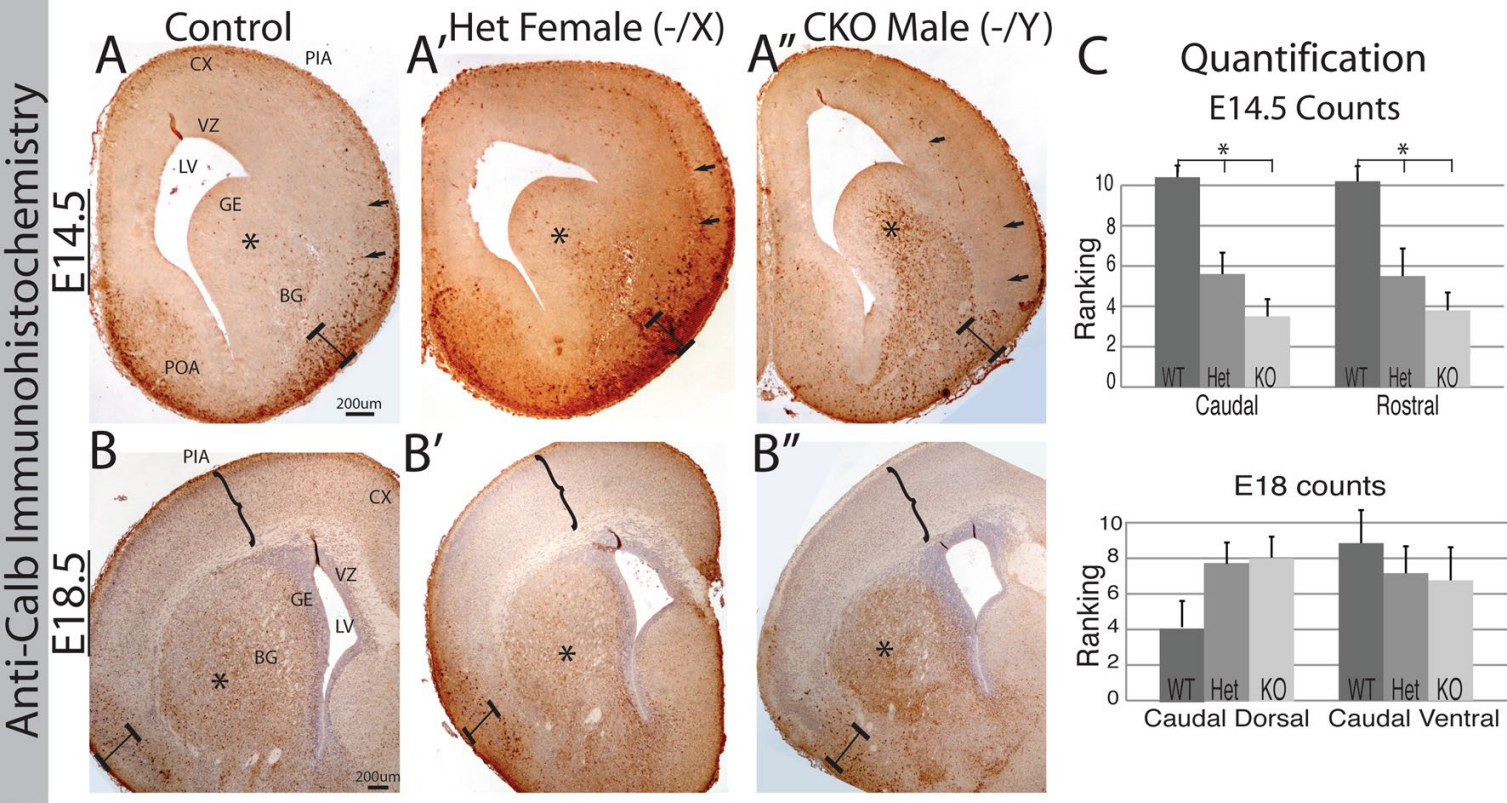
Calbindin immunohistochemistry at 2 embryonic time points reveals a shift in location of Calbindin positive cells. **A**–**A**″ *Upper row*, presents coronal sections at e14.5. In the control, Calb positive cells are present primarily ventrally in the POA and the developing amygdala complex (*bar* with ends) and sparsely in the cortex. In the mutants (**A**′–**A**″) there is an increase in expression in the SVZ of the GE (*asterisk*) and throughout the developing BG. A few cells still migrate into the cortex, though primarily in the IZ. **B**–**B**″ Lower row, presents coronal sections at e18.5. In the control, Calb positive cells are present primarily ventrally in the developing amygdala complex (*bar with ends*) and cortex (*brackets*), and sparsely in the BG (*asterisk*). In the mutants (**B**′–**B**″) there is an increase in expression in the BG (*asterisk*) and a significant loss in the cortex (*brackets*), and no changes in the developing amygdala (*bar with ends*). **C** Displays the quantification of Calbindin positive cell numbers from embryonic ages. All counts are presented as average ranking from rostral and caudal (and dorsal and ventral) sections at e14.5 (*upper*) and e18.5 (*lower*). *LV* lateral ventricle, *VZ* ventricular zone, *BG* basal ganglia, *Cx* developing cortex, *IZ* intermediate zone, *Hip* hippocampus. *L1* cortical layer 1, *L2/3* cortical layers 2 and 3, *L6* cortical layer 6

**Fig. 4:**
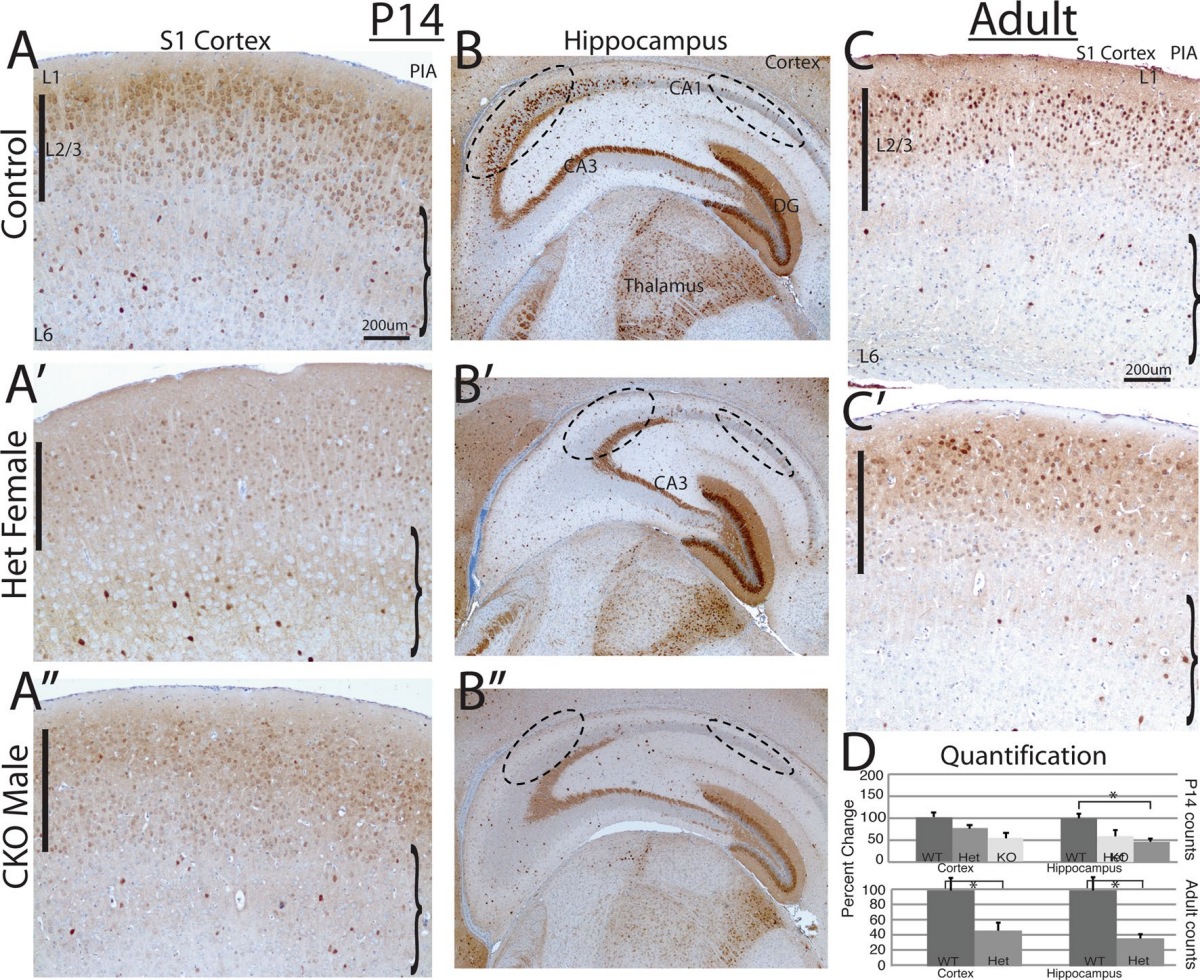
Calbindin immunohistochemistry at 2 postnatal time points revels a reduction of Calbindin positive cells. **A**–**A**″, **B**–**B**″ P14 left and middle column sections illustrates that Calb positive cells are present throughout the cortex and hippocampus, but with a reduction in the number of labeled cells. There was a trend toward more cells in the lower layers (*curved brackets*) but this did not reach significance. The hippocampus at P14, shows more significant changes (**B**–**B**″; *dashed circles* in CA1 and CA3 stratum radiatum). In the adult animal, (*right column*; **C**, **C**′) there is also a clear reduction in the number of Calb positive cells in the *Arx* ^+*/*−^ female (**C**′) versus control (**C**). **D** Displays the quantification of Calbindin positive cell numbers from post-natal ages. All adult section counts are presented as a percent change from control in both the cortex and hippocampus. *LV* lateral ventricle, *VZ* ventricular zone, *DG* dentate gyrus, *CA1* Cornus Ammonis 1, *CA3* Cornus Ammonis 3. *Pia* pia matter, *L1* cortical layer 1, *L2/3* cortical layers 2 and 3, *L6* cortical layer 6

Given the variable expression of Arx in the mature WT, *Arx*^+*/*−^, and *Arx*^−*/y*^ mouse brains, we next sought to define interneuron marker expression through development. Immunohistochemistry was performed for Calbindin, Calretinin, Somatostatin, NPY, and PV. The pattern of calbindin (calb) staining at e14.5 was clearly different among the WT, *Arx*^+*/*−^, and *Arx*^−*/y*^ mice. Compared to the WT, there was significantly more expression in the SVZ of the GE (Fig. 3A–A″, asterisk) as well as in the ventral telencephalon in the region of the ventral palladium and amygdaloid complex (Fig. 3A–A″, bar with ends). The increase ventrally is associated with a reduction of Calb positive cells in the dorsal telencephalon (Fig. 3A–A″, arrows). Observer ranking of the patterns of positive cells was significantly different in both the GE and cortex using both a whole caudal and rostral 4x images (Fig. 3C, p = 0.014 and 0.03, n = 6) and with focusing in on the ventral forebrain with 20× images of both rostral and caudal ventral regions (p 0.024 and 0.037, n = 6). The rankings indicate more positive staining in the *Arx*^−*/y*^ animals ventrally. The observer ranking of the number of positive cells dorsally in both the rostral and caudal regions was not significantly different (0.36 and 0.94, n = 6). At e18.5, no significant differences in Calb expression were noted (Fig. 3B–B″, c lower graph); however there was a trend in the rostral ventral sections showing more staining in the *Arx*^−*/y*^ animals (Fig. 3B, asterisk, square brackets, p = 0.07) but no clear difference in the dorsal cortex in this analysis. By P14, there is a significant loss of Calb cells in the hippocampus (Fig. 4, oval regions highlighting differences, p = 0.026). In the cortex, the number of Calb positive neurons was not significantly different among genotypes, however, there was considerable variability between sections (p = 0.37, WT—83.22 ± 10.7 vs *Arx*^−*/y*^—43.9 ± 19.01). We did note a prominent decrease of calb positive neurons in all regions counted in the Arx^−/Y^ brains. While it appeared there were fewer calb positive cells in the lower layers (brackets in Fig. 4A–A′), this again did not reach statistical significance. In adult *Arx*^−*/*+^ mice, despite considerable variability in the number of calb positive neurons, with some animals having a decrease and others no change, overall the mean was significantly different (Fig. 4C–C′, d; p = 0.013 in cortex and 0.01 in hippocampus).

**Fig. 5:**
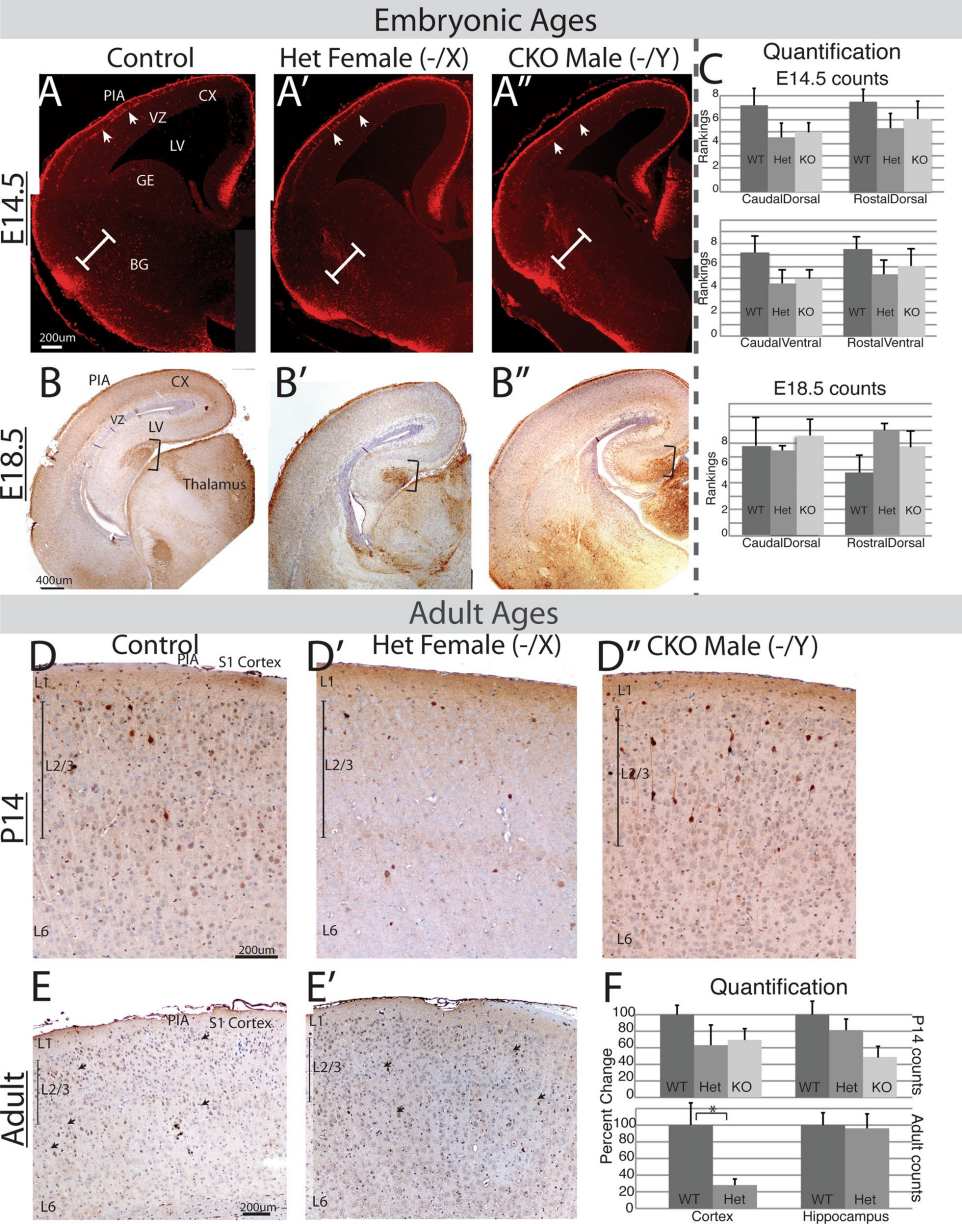
Calretinin immunohistochemistry at 2 embryonic and 2 post natal time points reveals alteration of expression across the ages. The *top row* (E14.5, **A**–**A**″) demonstrates effect of loss of *Arx* on *Calr* staining from representative e14.5 coronal images. There is an increase in the number of cells in the region of the developing internal capsule in the *Arx* ^−*/y*^ *;Dlx5/6* ^*CIG*^ compared to the wildtype (*bar with ends*) and the *Arx* ^+*/*−^ female has an intermediate number of labeled cells. In contrast, there is a decrease in the number of scattered cells throughout the ventral pallium. Dorsally, all three genotypes have a similar appearing clear migratory stream through the MZ/CP region (*arrows*). E18.5 (*second row*, **B**–**B**″) illustrates no major differences in Calr cells either ventrally or dorsally, with accumulating cells found in the hippocampus in all three genotypes (*bracket*). **C** Presents the quantification of the two embryonic time points. All counts are presented as average ranking from rostral and caudal (and *dorsal* and *ventral*) sections at e14.5 (*upper*) and e18.5 (*lower*). Adult Ages; P14 (*third row*; **D**–**D**″) sections illustrate that Calr positive cells are present throughout the cortex in control mice. There is a trend toward a reduction in the number of labeled cells in mutant mice but it does not reach significance (**F**). In the adult animal, (*bottom row*; **E**, **E**′) there is also a clear reduction in the number of Calr positive cells in the *Arx* ^+*/*−^ females (**E**′) versus control (**E**). **F** Displays the quantification of *Calr* positive cell numbers from adult animals. Counts are presented as a percent change from control in both the cortex and hippocampus. *LV* lateral ventricle, *VZ* ventricular zone, *BG* basal ganglia, *Cx* developing cortex, *Hip* hippocampus. *L1* cortical layer 1, *L2/3* cortical layers 2 and 3, *L6* cortical layer 6

Calretinin staining demonstrated a distinct pattern with no differences among wildtype and mutant animals found at embryonic ages by the ranking analysis (Fig. 5A–A″, B–B″, p = 0.36, 0.76 for rostral and caudal sections respectively) but with a region of staining in the ventral caudate and putamen in the *ARX*^−*/Y*^ and heterozygous females (Fig. 5A–A″, bar with ends) that was not well labeled in the wildtype. A trend towards loss of calretinin positive cells migrating in the developing cortical plate was observed across sections (Fig. 5A–A″ arrows), however this was not significant by the ranking method. At e18.5 and P14 there were no clear differences in *Arx*^−*/y*^ mice compared to controls (Fig. 5B–B″, C, D–D″, F; p = 0.2, 0.79 rostral and caudal sections, respectively). There also appeared to be an increase in calretinin positive cells in the dentate gyrus (DG) of the hippocampus (Fig. 5B–B″, square bracket), but this also did not reach significance with our ranking method (Fig. 5C, lower graph, p = 0.20). At P14, there was a trend towards significantly fewer cells in the hippocampus (p = 0.07), but no change in the cortex. There was a decrease in calretinin staining in the cortex of adult *Arx*^+*/*−^ mice (Fig. 5E–E′, F, p = 0.05) but not in the hippocampus (p = 0.87, Fig. 7F, lower graph).

**Fig. 6:**
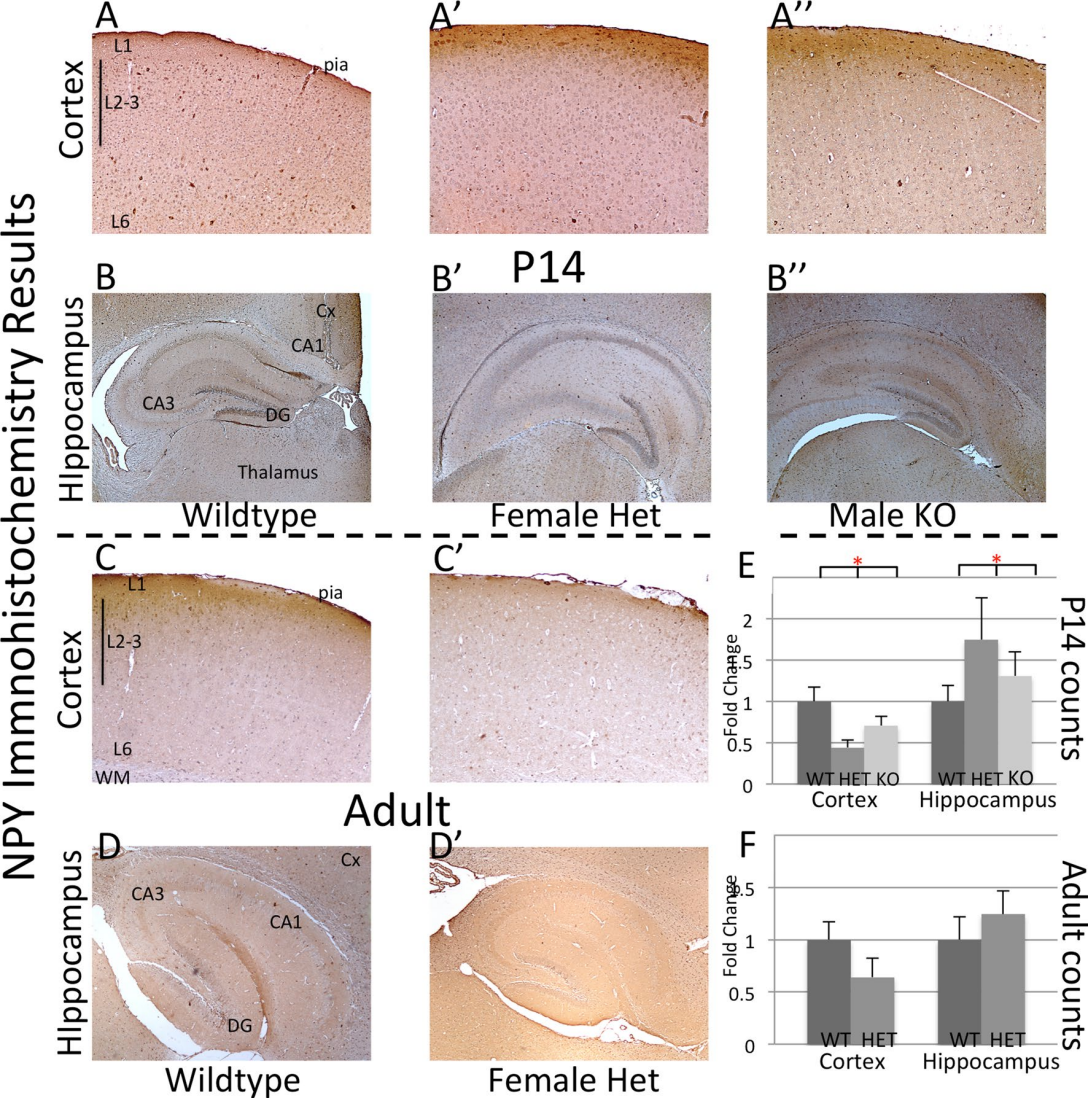
Neuropeptide Y immunohistochemistry at 2 postnatal time points revels alteration of expression at P14, but not adult. The *top row* (P14 cortex, **A**–**A**″) demonstrates a reduction of NPY positive cells in the cortex between the control and both *Arx* ^−*/y*^ *;Dlx5/6* ^*CIG*^ and *Arx* ^+*/*−^female animals. The *second row*, (P14 hippocampus, **B**–**B**″) illustrates a similar difference in NPY cells in the hippocampus as was found in the cortex. In the adult, there were no significant difference found between *Arx* ^+*/*−^female and wildtype in either the cortex (*third row*; **C**–**C**′) or hippocampus (*last row*, **D**–**D**′). **E**, **F** Displays the quantification of NPY positive cell numbers from P14 (**E**) and adult (**F**) measuring the percent change from control in both the cortex and hippocampus. *LV* lateral ventricle, *WM* white matter, *Cx* developing cortex, *Hip* Hippocampus. *L1* cortical layer 1, *L2/3* cortical layers 2 and 3, *L6* cortical layer 6, *DG* dentate gyrus, *CA1*–*CA3* Cornus Ammonis regions 3 and 1

In addition to using Arx, Calb and Calr as markers that we could track from prenatal to postnatal time points, we looked for changes in interneurons in older animals with immunohistochemistry for neural peptide Y (Npy), parvalbumin (Parv) and somatostatin (SOM). Immunohistochemical expression of Npy was absent at e14.5 in wildtype animals, corresponding to the in situ hybridization data from the Allen brain atlas (brain-map.org). At e18.5 there was a significant decrease in numbers of Npy positive cells in the ventral forebrain of the Arx^−/Y^ animals, with no difference dorsally (p = 0.02 and 0.07 in ventral rostral and caudal regions respectively and 0.83 and 0.15 in dorsal rostral and caudal regions). By P14, the loss of Npy cells in the cortex of the mutant animals persisted (Fig. 6A–A″, E; p = 0.039) but not in the hippocampus (Fig. 6B–B″, E; p = 0.32). In the heterozygous female adult animals there was no overall difference in either cortex or hippocampus, though a trend towards a decrease in the cortex was noted (Fig. 6C–C′, D–D′, E; p = 0.19).

For Somatostatin staining there was no expression in the embryonic ages by immunohistochemistry or in situ (from Allen Brain Atlas; brain-map.org) and in P14 and adult (female heterozygous only) there were no differences noted between the mutant and wildtype mouse brains (data not shown).

**Fig. 7:**
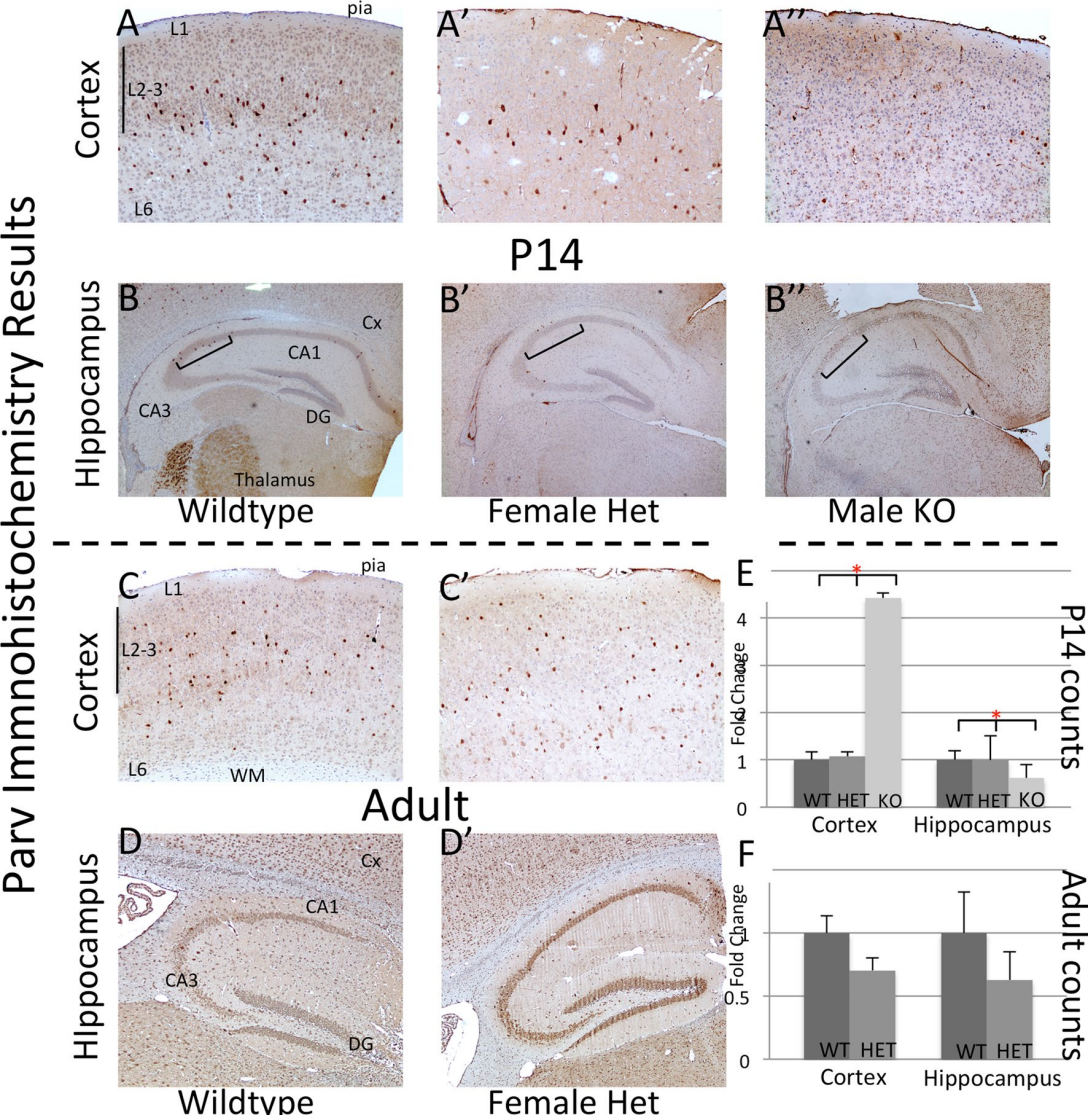
Parvalbumin immunohistochemistry at 2 postnatal time points revels altered expression patterns in hippocampus and cortex. *Top row* (P14 cortex, **A**–**A**″) demonstrates an increase in Parv positive cells in the cortex of *Arx* ^−*/y*^ *;Dlx5/6* ^*CIG*^ with no change in the *Arx* ^+*/*−^female animals. The hippocampus at P14 (*second row*, **B**–**B**″) reveals a consistent decrease in Parv expression in the *Arx* ^−*/y*^ *;Dlx5/6* ^*CIG*^ male but not *Arx* ^+*/*−^female. *Asterisks* denote significant differences. In the adult, there was a consistent trend toward a decrease between *Arx* ^+*/*−^female and wildtype male in both the cortex (*third row*; **C**–**C**′) and hippocampus (last row, **D**–**D**′). **E**, **F** Display the quantification of Parv positive cell numbers from P14 (**E**) and adult (**F**) measuring the percent change from control in both the cortex and ippocampus. *LV* lateral ventricle, *WM* white matter, *Cx* developing cortex, *Hip* hippocampus. *L1* cortical layer 1, *L2/3* cortical layers 2 and 3, *L6* cortical layer 6, *DG* dentate gyrus, *CA1–CA3* Cornus Ammonis regions 3 and 1

Lastly, the expression of *Parv* was compared between the wildtype and mutant animals. *Parv* is not expressed until P14 in the mouse (Brain-map.org). At P14 there was an interesting and paradoxical finding: a decrease in the Parv labeled neurons in the hippocampus of mutant males (Fig. 7B–B″, E; p = 0.019) but an increase in the cortex (Fig. 7A–A″, E; p = 0.0007, fourfold increase). In the heterozygous females there was a trend toward a decrease in the cortex and no change in the hippocampus (Fig. 7C–C′, D–D′, F; p = 0.17 and 0.46, respectively). As with Arx immunohistochemistry, Parv labeling showed considerable variability in females.

Overall, these results demonstrate that loss of *Arx* in the ventral pallium in the developing ganglionic eminences result in a shift of GE cells ventrally and a loss of the cells migrating into the dorsal forebrain. These changes ultimately lead to an early death in the males and a variable phenotype in the females as previously reported [2].

## Discussion

Conditional loss of *Arx* from the Dlx5/6 expressing population of neural progenitors results in an early postnatal death in males and results in survival but variable expression of *Arx* in females. From these observations we posited that mice with efficient deletion of *Arx* were dying due to a defect in inhibitory transmission and resultant neurological deficits. Our results indicate that cells in which *Arx* is lost still express the inhibitory marker *Gad67* but fail to normally migrate to the cortex. We observed a shift in the cells that expressed *Arx* to more ventral fates with expansion of the Calbindin and possibly Calretinin positive interneuron populations in these mice. At later time points, the expected loss of Calb, Npy, Sst, and Parv interneuron markers was observed. As we will discuss below, our data confirms the role of *Arx* in interneuron development and strengthens the evidence that *Arx* is not needed for expression of interneuron markers, rather *Arx* appears to be important for the normal migration of neurons from the GE into the cortex.

### The effect of Arx loss on interneuron populations and survival

The early lethality of mice with the interneuron-specific deletion of *Arx* underscores the importance of this gene. The phenotype of *Arx*^−*/Y*^*; Dlx5/6*^*CIG*^ mutant animals is in fact, more severe than we previously reported [2]. We had assessed the EEG background, presence of seizures, and interneuron populations in the adult male and female mice that we believed were due to a complete loss of function (*Arx*^−*/y*^). At the time, a working antibody directed against Arx was not available, and we based our findings on the presence of a recombined band on genotyping at early postnatal ages. Many of the animals with the recombined bands died shortly after birth and only 3 pups of 15 litters survived to adulthood [2]. The surviving *Arx*^−*/y*^ males are now presumed to have had significant persistence of *Arx* expression. In this study, we have shown variability of *Arx* expression (see below for discussion on Cre recombinase variability) that likely correlates with survival; animals with greater levels of Arx live longer.

Though humans do not have variable levels of expression due to cre recombinase activity, there is likely variability of protein function based on mutation type and genetic background. This is consistent with the genotype phenotype correlation found in humans with *ARX* mutations. Indeed, truncation mutations that result in loss of protein, or mutations in the homeodomain that abolish binding result in more severe XLAG phenotype, and these patients die early. On the other hand, missense mutations or expansion of the first or second polyalanine tracts in *ARX*, only cause early onset seizures and developmental delays, with the children surviving at least through childhood (for review of clinical features of *ARX* see [4, 6]). Previous reports on *Arx* have shown that these mutations do not cause Arx to lose repressor function in in vitro studies [30]. These less “severe” mutations could act by altering Arx activity in a subset of cells. In fact, we have found that *Arx* with an expansion of the first polyalanine tract leads to loss of repression of only a subset of targets [3]. Though mutation type can lead to a spectrum of conditions in humans and differing survival lengths in mice, these current studies confirm the importance of *Arx* for interneuron development and ultimate survival in the mice.

In these conditional mice with a more complete loss of *Arx* in the interneuron precursor population, we have found a loss of interneurons in the cortex and hippocampus at e18.5 and P14, and no survival of males with complete loss of *Arx*. This differs from findings in the two recently published mouse models of the expansion of the first polyalanine in *Arx* [10, 11]. Both expansion models had reductions in the numbers of striatal interneurons (both somatostatin- and Npy-expressing interneurons) with differing findings for cortical interneurons. Here at P14, we find a reduction of all interneurons in the hippocampus as well as the cortex (there was a paradoxical increase in PV cells in layer 1 that we believe is due to staining differences). We previously reported that the CKO males had loss of *Calb* and *Calr*, but we know that these were hypomorphs with substantial *Arx* expression (data not shown). Hence, in people with truncation mutations or homeodomain loss of function changes and a more severe phenotype, they likely have severe loss of interneurons, whereas there is likely only a minimal change in interneuron numbers in the mice (or people) with polyalanine tract expansions. This suggests that the seizures and intellectual issues in people and mice with an expansion mutation is not due to decreased numbers of interneurons, but rather a change in function, anatomy, or connectivity which may be related to the postnatal function of *Arx* in interneurons, a role that is unknown at this time.

### Increased calbindin in subpallium and regulation of striatal interneuron migration

The increased number of calbindin-expressing neurons in the striatum of *Arx*^−*/Y*^*; Dlx5/6*^*CIG*^ mice suggests that *Arx* regulates one or more migratory guidance factors. The striatum consists of patch and matrix compartments. The patch compartment, also known as striosomes, contains Darpp32+ medium spiny neurons, while calbindin normally labels projection neurons receiving input from cortical areas in the matrix compartment of the striatum [31, 32]. In agreement with our immunohistochemical results on the *Arx*^−*/Y*^*; Dlx5/6*^*CIG*^ mice, a microarray study of subpallial brain tissue from the conditional deletion of *Arx* from the forebrain of the developing mouse showed that calbindin is upregulated at e14.5 in the absence of *Arx* [1, 33]. Normally, the neuropilin receptors are involved in repelling interneurons from the subpallium toward the pallium [34]. For example, expression of a dominant negative Neuropilin-1 allows interneurons to aberrantly enter the striatum [35]. However, the effects of the neuropilins are not clear, as down-regulation of Neuropilin-2 seems to yield contrasting results. In one case, it has been found that *Nkx2.1* directly represses Neuropilin-2 in post-mitotic interneurons, by allowing them to migrate into the striatum, which normally repels cortical-bound interneurons through Neuropilin-2 receptor interactions with Semaphorin-3F [36]. On the other hand, *Dlx1/2* have also been found to repress Neuropilin-2 in interneurons that migrate dorsally into the cortex, which may allow them to pass through the Semaphorin-expressing striatal area, as areas of Neuropilin-2-expressing ectopia are found in the SVZ of the LGE and MGE of *Dlx1/2* mutant embryos [37]. Although Neuropilin-1 was found to be up-regulated in a microarray study on the *Arx* mutant [1], this is not likely sufficient to explain the increase of calbindin-expressing interneurons in the striatum of *Arx*^−*/Y*^; *Dlx5/6*^*CIG*^ mice, because the cells were found evenly dispersed throughout the striatum, rather than clustered in ectopia. The complexity of ventral repellant and attractant forces upon interneurons is demonstrated by the *Ctip2*^−*/*−^ mouse, which has clusters of ectopic cells expressing elevated levels of Neuropilin-1 despite no loss of striatal semaphorin expression [38]. Therefore, some other signals are capable of overriding the repellant activity of the semaphorins. One known component of this complexity is the Slit/Robo interaction, which is another pathway that has been hypothesized to be involved in repelling cortical-bound interneurons toward their dorsal route [39, 40]. Increased numbers of calbindin-positive cells are seen in both the cortex and striatum of *Robo*^−*/*−^ mice [40].

### The effect of heterozygous Arx expression

The EEGs of the adult *Arx*^−*/X*^*; Dlx5/6*^*CIG*^ female mice showed great variation: mice either had normal background recordings or interruptions of chaotic activity, and 53 % of the females had seizures [2]. In females with a normal copy of *Arx*, there is an incomplete loss of *Arx* in *Dlx5/6*-expressing populations of interneurons. This incomplete loss results in the survival of all the female animals and with varying degrees of neurological manifestations. This incomplete loss of *Arx,* still was detrimental enough to result in a change in the profile of interneurons in the adult females, including a reduction of the calbindin and calretinin expressing subpopulations but only trends in the *Parv* and *Npy* populations. This heterozygous loss contrasts to the complete loss in males where only the extremely rare male with poor recombination survived. The variability in the heterozygous female animals and in X-linked recessive disorders in people is often attributed to skewing of X-inactivation from the predicted 50 % suppression of each chromosome to the situation of either unexpectedly severe or mild phenotype in an X-linked disease. Here we clearly demonstrate the variability of X-inactivation.

Previously, we studied the role of X-inactivation in the phenotype of the *Arx*^−*/X*^*; Dlx5/6*^*CIG*^ mice, and could not rule out a subtle contribution of X-inactivation to the variability of the phenotype seen in females with *ARX* mutations [2]. In the *Arx*^−*/X*^*; Dlx5/6*^*CIG*^ mice that had efficient Cre-mediated recombination, a striated pattern of *Arx* expression resulted. Although, in general, large areas of the subpallium continued to express *Arx*, the amount of *Arx* expression, presumably due to X-inactivation, varied from animal to animal. Also of note is how early the *Dlx5/6* enhancer could be activated. Frequently, striations of *Arx*-negative tissue extended from the ventricular zone, through the subventricular zone, out into the mantle zone, suggesting that X inactivation occurs in the ventricular zone. While this has been demonstrated in the dorsal VZ and in cerebellum, it has not previously been shown in the ventral VZ [27, 41].

### Heterogeneity in Dlx5/6 enhancer-driven recombination

We observed an unexpectedly widespread expression of *Arx* in Dlx5/6-expressing populations of neurons in *Arx*^−*/Y*^*; Dlx5/6*^*CIG*^ mice. The *Dlx5/6*^*CIG*^ mouse has been widely used [42–44], but the animal-to-animal variation in its Cre-mediated recombination has not been investigated. In previous studies, deletion by loxP recombination has been measured by Western blot or PCR, in which each reaction represents at least an individual animal, if not multiple animals. One study of Cre-mediated genomic recombination found that the Cre driver line recombined the responder loxP sites in 46, 72, 76 and 93 % of animals, for 4 different responder lines, respectively [45]. Recombination was determined by PCR, which does not indicate the efficiency of recombination within the animal, on a cell-by-cell basis. However, the authors hypothesize that the efficiency they calculated was dependent, at least in part, on the accessibility of the chromatin structure at the integration site [45]. The issue of variably Cre mediate recombination, has indeed been used to achieve low recombination efficiency to permit study of the morphology of scattered cells. One group found that efficiency of recombination within one animal could vary by approximately one thousand-fold depending on chromosomal location, target gene sequence and developmental age. The latter differences believed to be due to epigenetic changes in gene structure [46]. Overall, the variability of expression in males as well as X-inactivation in females results in a more challenging interpretation of the loss of the genes effect in both the normal and disease states.

### Role of Arx in interneuron development

This phenotypic description of the changes in interneuron development highlights the importance of *Arx* in cortical interneuron development. This work suggests that the main function for *Arx* is the transcriptional control of cues or the machinery to enable migration from the ventral forebrain into the cortex. Our previous study, which profiled the changes in expression in the ventral forebrain after *Arx* loss demonstrated that genes involved in migration and axonal guidance were most dysregulated [1]. This was confirmed with work that demonstrated that *Arx* was downstream of the Dlx1/2 transcription factors and involved in migration [22]. While this work does not further the molecular underpinnings of this shift in migration, it demonstrates that there is a ventral shift in migration of interneuron precursors and that there is not a loss or shift in fate of these cells.

## Conclusion

In summary, we have found that loss of *Arx* in ventral progenitors leads to perinatal lethality in mice and appears to shift the progenitors ventrally and decrease the dorsal migration of these precursors. The end result is a reduction of interneurons in the cortex in the *Arx*^−*/X*^*; Dlx5/6*^*CIG*^ mouse mice in perinatal and early postnatal time periods. In addition, we show that X-inactivation results in striated patterns of expression in the heterozygous females providing strong evidence that variability in X-inactivation can explain the variable phenotype found in human females with *ARX* mutations. These finding continue to shed light on the role of *Arx* in ventral neural progenitor function and the spectrum of *Arx* related neurological disorders.

## Abbreviations

*ARX/Arx* (*human/mouse*): aristaless related homeobox gene
ID: intellectual disabilities
XLAG: X linked lissencephaly with ambiguous genitalia
IRES: internal ribosomal entry site
GFP: green florescent protien
PBS: phosphate buffered saline
PFA: paraformaldehyde
ANOVA: analysis of variance
ROI: region of interest
e14.5/e18.5: embryonic day 14.5/18.5
P14: postnatal day 14
GE: ganglionic eminence
SVZ: subventricular zone
CP: cortical plate
IZ: intermediate zone
WT: wild type
N: number
PV: parvalbumin
NPY: neuropeptide Y
Calb: calbindin
DG: dentate gyrus
SOM: somatastatin
CKO: conditional knock out
